# New Appliances and Things Medical

**Published:** 1893-05-20

**Authors:** 


### NEW APPLIANCES AND THINGS MEDICAL.

[All preparations, appliances, novelties, etc., of which a notice is desired, should be sent for the Editor, to care of The Manager, 428, Strand, London, W.C.]

### DR. LANGEN'S SUGAR OF MILK.

(Burroughs Brothers of 66, Basinghall Street, E.C.)

We have received a tin of the above. It is an excellent specimen, in powder, of what it claims to be. In making the usual dilution of cow's milk for babies it is customary to use ordinary cane sugar to sweeten the mixture, but it stands to reason that the natural sugar of milk would be more useful. We note that the directions state that the milk to be diluted should be boiled, and also the solution of the sugar. Where the milk supply, as of necessity it almost must be in town, is open to aerial contamination, this is the best method of preparing it. The sugar of milk will also form a useful addendum to the units of nutrition for the aged and invalid. We wonder why it has not before been more extensively used?

